# Quantitative analysis of mucociliary activity by computational high-speed video reflection microscopy

**DOI:** 10.1007/s00249-026-01835-6

**Published:** 2026-03-16

**Authors:** Martin Schneiter, Julian Wyss, Jeremy Schori, Patrik Arnold, Martin Frenz, Pietro Cicuta, Stefan A. Tschanz, André Stefanov, Loretta Müller

**Affiliations:** 1 Institute of Applied Physics, University of Bern, Bern, Switzerland; 2 Institute of Anatomy, University of Bern, Bern, Switzerland; 3 Bern University of Applied Sciences, Engineering and Information Technology, Biel, Switzerland; 4 Cavendish Laboratory, University of Cambridge, Cambridge, UK; 5 Division of Paediatric Respiratory Medicine and Allergology, Department of Paediatrics, Inselspital, Bern University Hospital, University of Bern, Bern, Switzerland; 6 Lung Precision Medicine (LPM), Department for BioMedical Research (DBMR), University of Bern, Bern, Switzerland

**Keywords:** Cilialyzer, primary ciliary dyskinesia (PCD), ciliary beat frequency (CBF), mucociliary clearance (MCC), air-liquid interface (ALI)

## Abstract

The inner surface of our airways is lined by a mucous fluid film that is continuously propelled towards the throat. The propulsion of this airway surface liquid is generated by the collectively coordinated oscillatory motion of a myriad of subjacent motile cilia. Inhaled particles are entrapped by the mucus layer and transported towards the pharynx where they are swallowed. Therefore, mucociliary clearance contributes to our airway’s primary defense mechanism by protecting our airways from inhaled particles. We developed a computational high-speed video reflection microscopy (CVRM) system that enables imaging and quantitative characterization of the mucociliary activity – particularly in terms of the cilia-caused dynamic modulation of the mucus surface – in air-liquid interface cell cultures. Nasal epithelial cells from healthy volunteers and people with primary ciliary dyskinesia (PCD) were differentiated at the air-liquid interface, representing a sophisticated model widely used to study the mucociliary clearance mechanism. The cell cultures were imaged by reflection microscopy using a high-speed camera and the videos were quantitatively analyzed using a newly extended version of our Cilialyzer software. The primary aim of this study was to develop and validate CVRM as a quantitative method for characterizing collective mucociliary dynamics under physiological air–liquid interface conditions. We found that the dynamic modulations of the mucus surface closely correspond to the underlying ciliary motion. By using specifically developed video processing methods, we quantitatively characterized the collective mucociliary activity in terms of ten space-time features. This allowed us to distinguish cell cultures derived from people with PCD from those derived from healthy volunteers and demonstrates that CVRM has clear potential for future diagnostic applications in PCD and beyond.

**Supplementary Information:**

The online version contains supplementary material available at https://doi.org/10.1007/s00249-026-01835-6.

## Introduction

Mucociliary clearance represents the primary defense mechanism of the human respiratory system and refers to its innate ability of self-cleaning. When we breathe, we inhale toxic and potentially infectious particulate matter, which is entrapped by a mucous fluid film lining the inner surface of our airways. This so-called airway surface liquid is permanently transported towards the throat, where the mucus-embedded particles are expectorated or swallowed. The propulsion of the airway surface liquid relies on the coordinated activity of countless subjacent cilia, which densely populate the luminal epithelium of our airways. The importance of effective mucociliary clearance is particularly highlighted by its impairment in respiratory diseases such as primary ciliary dyskinesia (PCD), cystic fibrosis, asthma and chronic obstructive pulmonary disease. PCD refers to a genetically and clinically heterogeneous disorder of ciliary biogenesis, structure and function. It is predominantly characterized by chronic respiratory tract infections resulting from an insufficient (or absent) mucociliary clearance.

To this day, accurately measuring the effectiveness of mucociliary clearance in vivo remains challenging (Marthin et al. 2023; Sher et al. 2024). Moreover, from a diagnostic point of view, in vivo assessments of mucociliary transport are not always informative, as secondary effects may significantly impact the measurements. In PCD diagnostics, the primary efficacy of a patient’s mucociliary airway clearance is presently inferred from a combination of sophisticated diagnostic tests probing the function and structure of cilia (Müller et al. 2021). Secondary effects can be avoided by growing primary airway cell cultures, which re-differentiate into a fully functional respiratory epithelium exhibiting mucociliary transport and thus allowing us to examine the primary mucociliary clearance mechanism in vitro (Heguy et al. 2003; Sears et al. 2015).

In PCD diagnostics, transmitted-light microscopes are typically used to perform high-speed video microscopy (HSVM) allowing for the direct observation of the motion of cilia in freshly brushed nasal epithelial cells or in cells scratched off from air-liquid interface (ALI) cell cultures (Bricmont et al. 2021; Chioccioli et al. 2019; Kempeneers et al. 2019; Rubbo et al. 2019). However, in both cases the cells are immersed in a culture medium representing conditions that greatly differ from the native conditions where the air-exposed viscoelastic mucus layer is propelled by the underlying collectively coordinated ciliary activity of large continuous arrays of ciliated cells. The conventional HSVM analysis currently relies on the visual recognition of clearly discernible defects in the motion of cilia, which requires extensive empirical experience and is a time-consuming as well as subjective process (Bricmont et al. 2021; Kempeneers et al. 2019; Shapiro et al. 2020).

The growth in computing power over recent decades has led to the development of computational methods to quantitatively characterize the motion of cilia. The development can roughly be divided into two main lines of research. The first focuses on computational methods for the quantitative, observer-independent, and automated characterization of the motion pattern of individual cilia from detached, submerged airway epithelial cells (Bricmont et al. 2021; Chioccioli et al. 2019; Kim et al. 2011; Meste et al. 2015; Papon et al. 2012; Puybareau^2016^, ^2015^; Quinn 2011; Quinn et al. 2015; Zain 2020).

From a wider perspective, the reliability of the conventionally performed HSVM analysis approach is inherently limited, as detached ciliated cells being immersed in culture medium can only partly reflect the quality of the primary mucociliary clearance mechanism. The mucociliary clearance mechanism is an excellent example of a complex dynamical system with a local-to-global character, as the local fluid mechanical interactions of oscillating individual cilia are thought to generate the collective motion pattern, which eventually generates efficient mucociliary transport (Brumley et al. 2014; Elgeti and Gompper 2013; Schneiter et al. 2020). How the characteristic motion pattern of individual cilia is related to the collective motion pattern of cilia is still not fully understood, but it can be presumed that small aberrations in the individual ciliary motion pattern will amplify due to local fluid mechanical interactions and be more clearly reflected by the collective motion pattern as exhibited by large intact arrays of ciliated cells (Cicuta 2020). This presumption is tightly connected to the second line of research, which aims to investigate and quantify the collective mucociliary phenomena in human respiratory cell cultures, which are grown and observed at the ALI (e.g. see (Feriani et al. 2017; Khelloufi et al. 2018; Sears et al. 2011, 2013, 2015). From a physical point of view, ALI cell cultures match the natural conditions closely, as they present the collective mucociliary activity of thousands of ciliated cells covered by an air-exposed viscoelastic mucus layer (e.g. see Jory et al. 2019). Recently, an in-vitro essay for cilia-driven fluid flow in ALI cell cultures has been proposed as a diagnostic readout for PCD (Biebach et al. 2022; Loges et al. 2024; Wohlgemuth et al. 2024). It is important to note that the proposed test indeed provides a functional readout, but due to the addition of liquid volumes from the apical side, it does not replicate physiological ALI conditions and therefore does not reflect actual mucociliary clearance.

In this study, we demonstrate that reflection microscopy, which exploits the light reflected from the dynamically modulated air-exposed surface of the mucus layer – whose modulation is caused by the collectively coordinated motion of underlying cilia – can effectively image the collective mucociliary activity and offers highly favorable imaging properties. By processing high-speed videos with an extended Cilialyzer software (Schneiter et al. 2023), we can determine several physical observables quantitatively describing the space-time features of the collective mucociliary activity. We particularly demonstrate that multivariate discriminant analysis using computational high-speed video reflection microscopy (CVRM) effectively distinguishes between different phenotypic profiles of mucociliary activity associated with distinct underlying PCD genotypes. This highlights the method’s strong potential for detecting changes in mucociliary activity, as exemplified here by PCD, and suggests its future applicability for PCD diagnostics and other applications aimed at detecting subtle changes in mucociliary function.

## Methods

### Sample preparation

**Fig. 1:**
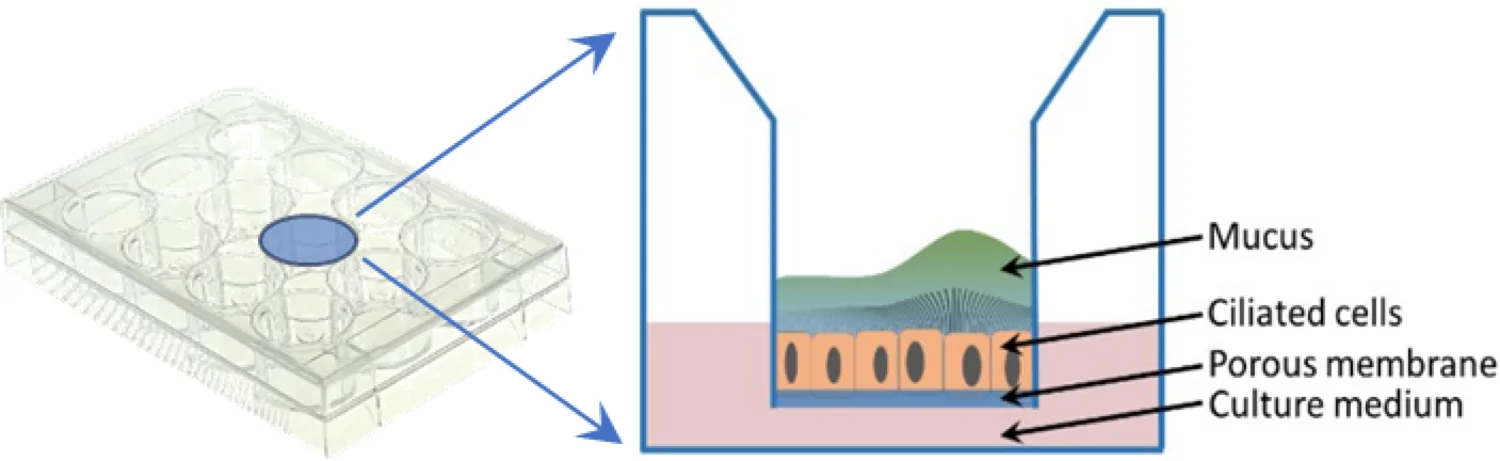
Transwell inserts (right) were kept in closed 12-well plates (left) during measurement

Nasal epithelial cells were obtained by nasal brushings and expanded in PneumaCult Expansion Plus medium (Stemcell Technologies) (for details see (Müller et al. 2021)). After reaching confluence in 12-well inserts (0.4 μm pore size, Celltreat, Stemcell Technologies), cells were cultured at the air–liquid interface with PneumaCult ALI medium (Stemcell Technologies) for four weeks until mucus production and ciliary beating were observed. Before measurements, cells were washed with PBS containing Mg²⁺ and Ca²⁺ for 30 min at 37 °C to remove excess mucus, and basolateral medium was replaced with Hepes-buffered PneumaCult ALI medium. Experiments were conducted on intact cultures kept in closed plates to maintain humidity (Fig. 1). Imaging was performed 24–48 h after washing if not indicated differently. Transient increases in ciliary beating frequency (CBF) and metachronal wavelength, and reduced mucus reflectivity occurred shortly after washing due to temporary mucus thinning (see Sect.  1 of the online supplement for details).

The study was approved by the ethics committee of the canton of Bern, Switzerland (ref. no. 2018–02155), and written informed consent was obtained from all participants or their legal guardians.

### Microscopic imaging: custom-built research and commercial system

**Fig. 2:**
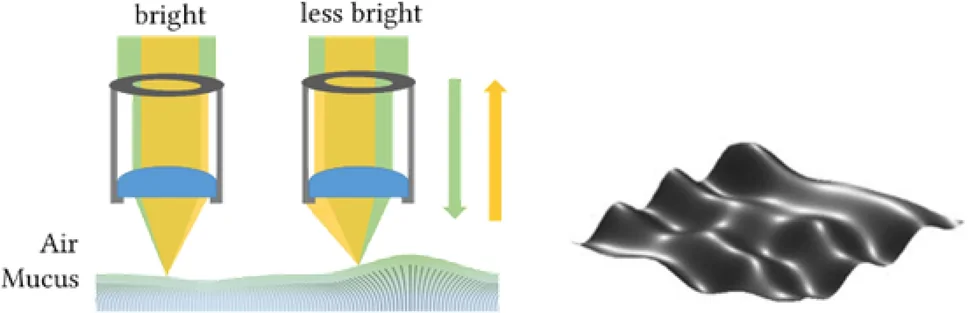
Illustration of the basic underlying principle of reflectometrically imaging the cilia-caused modulation of the overlying mucus surface: flat surface spots reflect more light back into the objective lens (left) and steeper spots reflect less light back into the objective (right). Consequently, inclined spots appear darker than flat ones, as illustrated by the modulated surface on the right

We employed reflection microscopy to optically image the collective mucociliary activity by imaging the local slope to the optical axis of each point on the mucus surface (Fig. 2). The mucus modulations are caused by the coordinated motion of the cilia underneath the mucus layer. Their motion constantly shapes and shifts the mucus layer, leading to changing light patterns and the variation in brightness (greyscale values) seen on the camera.

**Fig. 3:**
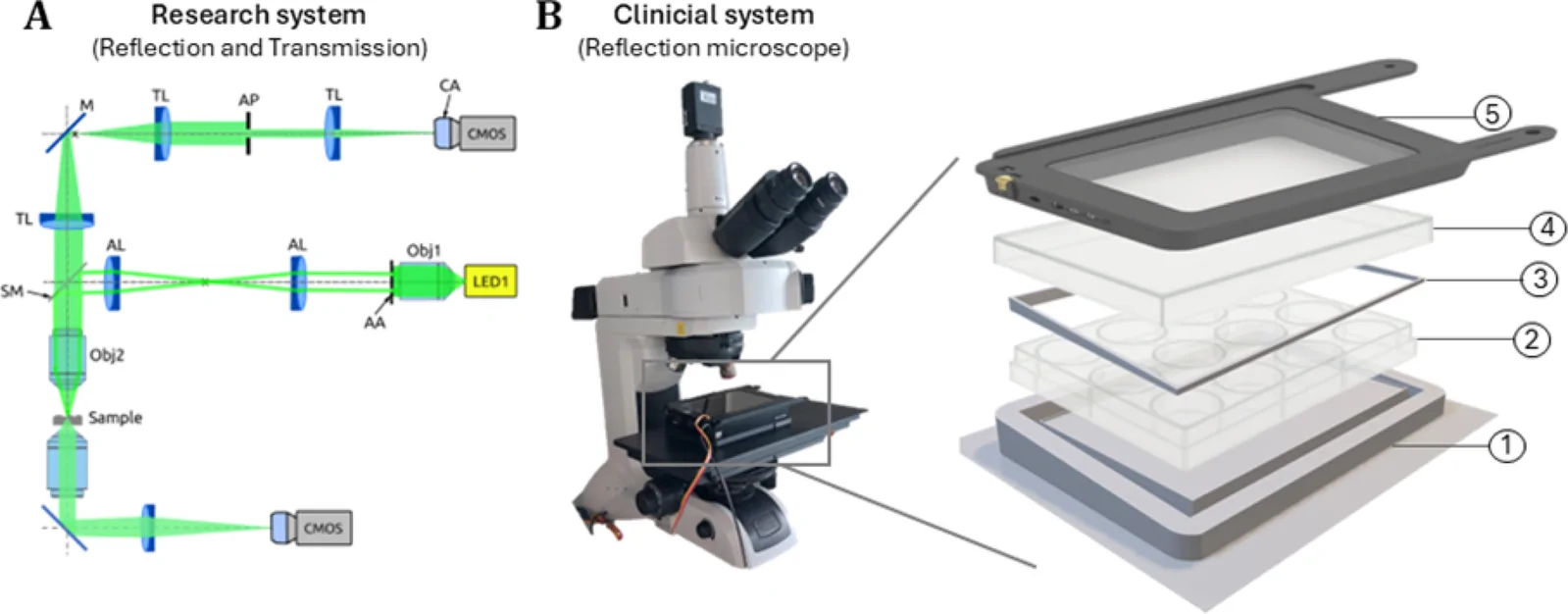
The presented data was collected with two different high-speed video microscopy systems. (**A**) custom-built research system that enables imaging in both reflection and transmission mode. (**B**) commercially available reflection microscopy system allowing for various clinical applications. The exploded-view shows: (**1**) 3D-printed component tilting the temperature-controlled frame from Okolab to prevent collecting light reflected from the incubator’s lid, (**2**) Bottom of transwell plate, (**3**) Ring-shaped 3D-printed component to tilt the lid of the transwell plate, (**4**) lid of transwell plate, (**5**) Temperature-controlled frame from Okolab. Abbreviations: AA=annular aperture, AL=achromatic lenses, AP=aperture, CA=camera, CMOS=Complementary Metal-Oxide-Semiconductor, Obj=objective, SM=splitting mirror, TL=tube lenses

For the present work, two high-speed video reflection microscopy systems were used (Fig. 3):

**Custom-built research microscope system**: The custom-built research microscope system combines reflected- and transmitted-light microscopy and uses a high-power white LED as the light source of a critical illumination configuration (Fig. 3A). The emitted light passes through an objective (Obj1: Nikon, CFI Plan Fluor 10x objective, NA 0.3, WD 16 mm) equipped with an annular aperture producing a collimated annular beam. The ring-like aperture gets imaged into the back focal plane of the observation objective (Obj2: Nikon, CFI Achro 4x objective, NA 0.1, WD 30 mm) by passing two identical achromatic lenses and getting partially reflected by a splitting mirror. The observation objective forms a hollow light cone, which is focused on the sample surface. The beam path for the back-scattered light is then inverted by two tube lenses to produce an intermediate field plane, which is equivalent to the back focal plane of the observation objective. In this Fourier plane, an adjustable aperture acts as a spatial filter to set an adequate contrast. Finally, the image is formed by the last tube lens and scaled down by a camera adapter lens (CA: Nikon C-0.45x tv lens), which is attached to a digital high-speed camera (FLIR, monochrome Blackfly S, BFS-U3-32S4M-C USB 3.1). The transmitted light is collected by an additional observation objective (40x, NA 0.6, WD 3 mm). With another tube lens and camera adapter, the motion of the cilia is projected in brightfield contrast on the sensor of an additional high-speed camera (FLIR, monochrome Oryx, ORX-10GS-32S4M-C). Most measurements were taken with the inserts in closed plates placed in a custom-built, temperature-controlled (37 °C) chamber surrounding the microscope stage of an upright microscope.

#### Commercial microscope system

The commercial microscope system is based on an upright Nikon microscope (Eclipse LV100ND, #MBA61135). Epi-illumination was delivered by a Nikon LV-LL LED lamphouse (#MBE65700). Using a Nikon CFI Plan Achromat 4× objective (WD 30 mm, NA 0.1, Nikon #MRL00042) a field of view of 544 × 408 µm^2^ was imaged onto 640 × 480 pixels of a high-speed camera (Grasshopper 3 GS3-U3-32S4M-C, FLIR). Recordings were acquired at 300FPS for 3 s. During the measurements, inserts were kept in closed plates placed in a temperature-controlled stage-top incubator (Okolab). Since both the plate lid and the lid of the stage‑top incubator reflect light and would otherwise disturb the reflected image, the Okolab stage‑top incubator was modified as illustrated by the exploded-view (Fig. 3B). To prevent these reflections from interfering, both lids were tilted by an angle $\alpha\approx{\mathrm{sin}}^{-1}\left(NA=0.1\right)$ relative to the plane perpendicular to the optical axis (for our setting 5.74°). This was achieved using two custom-designed, 3D‑printed PLA parts. Part 1 tilts the frame of the chamber, while Part 3 tilts the plate lid (Fig. 3B). Note that Part 1 is designed so that, although the chamber lid is tilted, the mucus surface remains horizontal (i.e., perpendicular to the optical axis).

#### Recordings of mucociliary dynamics and mucociliary transport

Recordings of the mucociliary dynamics, represented by the collective beating pattern of the cilia, were captured for 3 s at 300 frames per second (FPS) with maximum contrast. This is typically the case when the mucus layer covering the cilia is rather thin. In contrast, mucus transport can be observed most effectively in areas with a thicker mucus layer. Those recordings were acquired over 5 to 10 s at 10 to 30 FPS with 640 × 480 pixels.

### Video processing

The newest version of the Cilialyzer software and information on future updates will be made available at: https://msdev87.github.io/Cilialyzer/.

#### Automated analysis pipeline for videos showing mucociliary dynamics

**Fig. 4:**
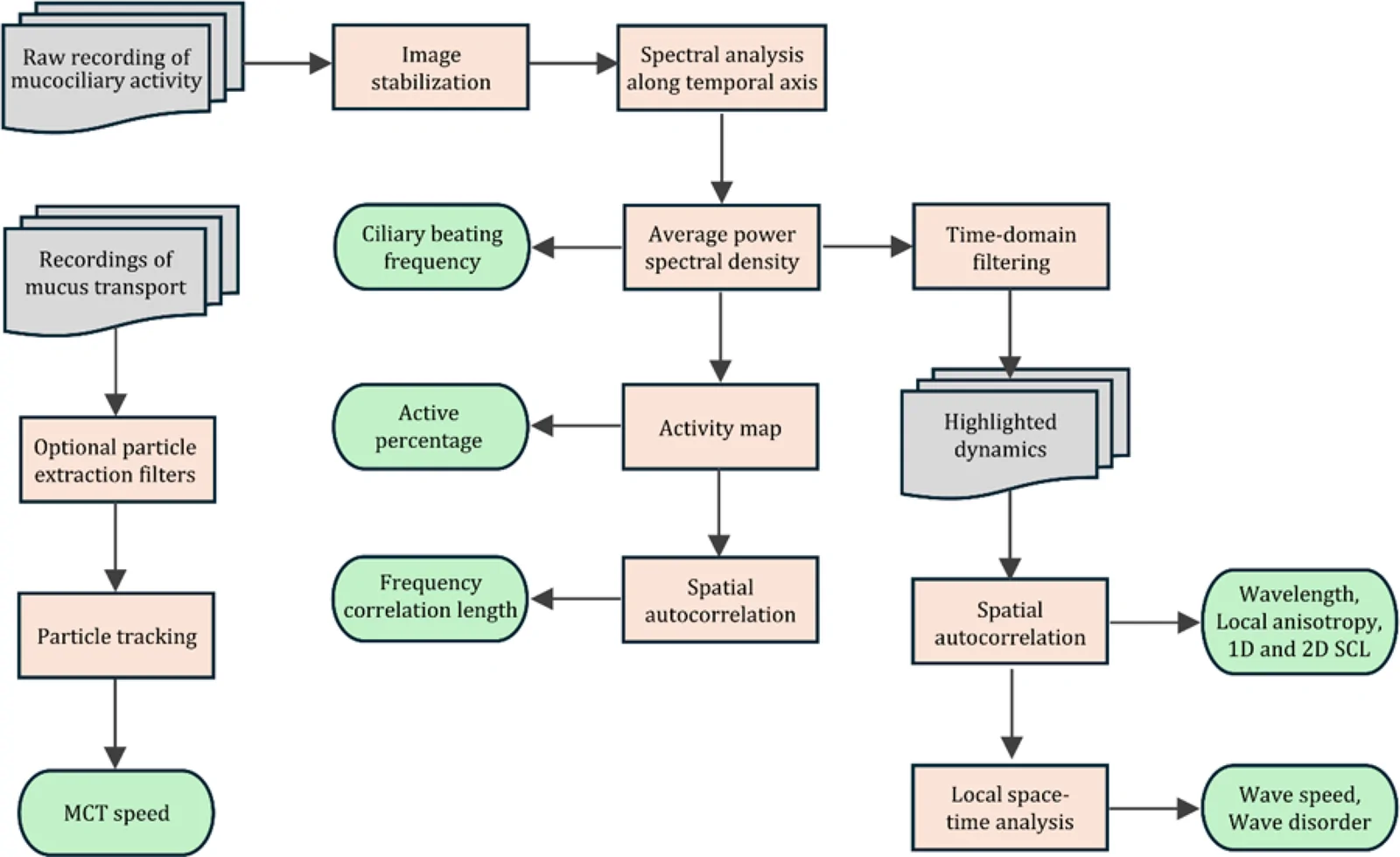
Flowchart outlining the processing of the videos, separated for transport recordings and recordings showing the mucociliary dynamics. Gray-shaded boxes represent image sequences, orange boxes represent processing steps, and green boxes represent observables or features that quantitatively characterize mucociliary activity. Abbreviations: MCT=mucociliary transport, SCL=spatial autocorrelation length

Videos showing mucociliary dynamics were analyzed using an automated analysis pipeline. By using an example video (Video S1, original; Video S2, preprocessed), we illustrate the determination and significance of each computed quantitative observable (overview in Table 1, flow chart in Fig. 4). To maintain clarity, only simplified explanations are presented here. More detailed algorithmic and mathematical formulations can be found in the Online Supplement.

**Table 1:** Overview of the computed set of observables/parameters characterizing the mucociliary activity in ALI cell cultures

Observable	Symbol	Unit	Qualitative meaning
Mucociliary transport (MCT) speed	v_T_	*μ*m/s	Transport speed of the mucus layer
Average ciliary beating frequency (CBF)	*ν*	[Hz]	Average motion frequency of cilia
Active percentage	*α*	[%]	Percentage of the field of view for which ciliary activity is detected
Frequency correlation length	${\xi}_{\nu}$	[*μ*m]	Indicator for distance over which the CBF is roughly maintained
Metachronal wavelength	*λ*	[*μ*m]	Spatial periodicity of the collective ciliary motion pattern
1D Spatial correlation length	${\xi}_{1\mathrm{D}}$	[*μ*m]	Indicator for distance over which the coordination is maintained
2D Spatial correlation length	${\xi}_{2D}$	[*μ*m]	Indicator for patch size within which the coordination is maintained
Anisotropy ratio	*ζ*	-	Quantifies the directional coherence of the collective motion of cilia
Average wave speed	v_w_	*μ*m/s	Average propagation speed of the wavelets
Wave disorder	*γ*	-	Variance of the local wave propagation direction

#### Ciliary beating frequency (CBF) ($\boldsymbol{\nu}$)

**Fig. 5:**
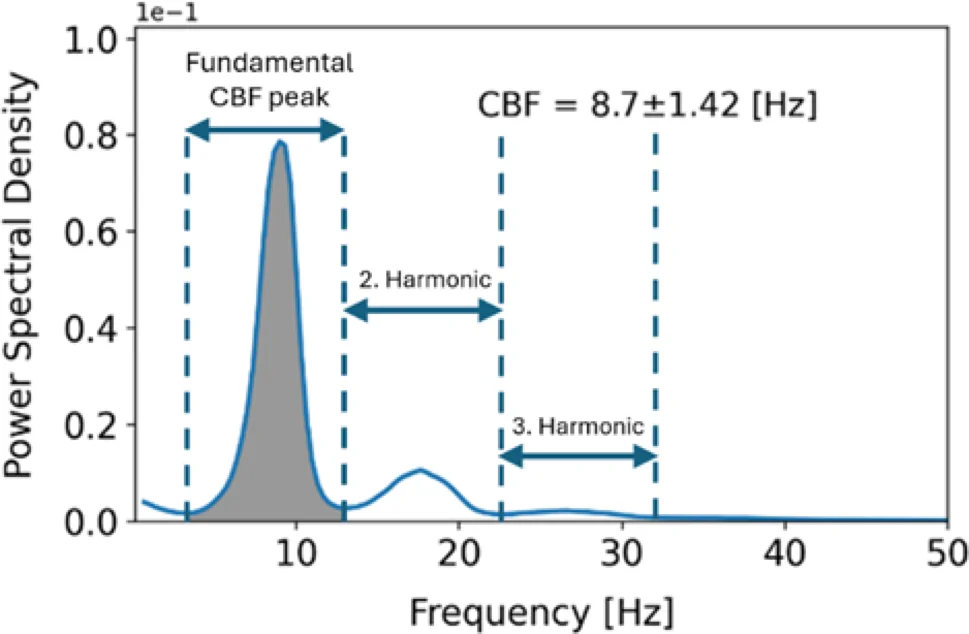
Average relative power spectral densitiy (PSD) – obtained by averaging each pixel’s temporal relative PSD. The ciliary beating frequency (CBF) is determined as the PSD-weighted average within the fundamental CBF peak (indicated by the grey shaded area)

The **CBF (**$\boldsymbol{\nu}$**)** corresponds to the average beating frequency of the cilia captured in the video. In simple terms, the temporal relative power spectral density (PSD) is first computed for each pixel. Subsequently, the area-averaged PSD is obtained by averaging over the entire field of view. This area-averaged PSD provides the weighting used to determine the CBF, which is calculated as the weighted average within the fundamental frequency peak (Fig. 5). The area-averaged PSD exhibits higher harmonics of the fundamental ciliary oscillations (Fig. 5), which are visible as less prominent peaks at integer multiples of the CBF and are present because the surface oscillations are not purely harmonic. Please refer to Online Supplement Sect.  2.1 for further details on the CBF algorithm.

#### Active percentage (*α*)

**Fig. 6:**
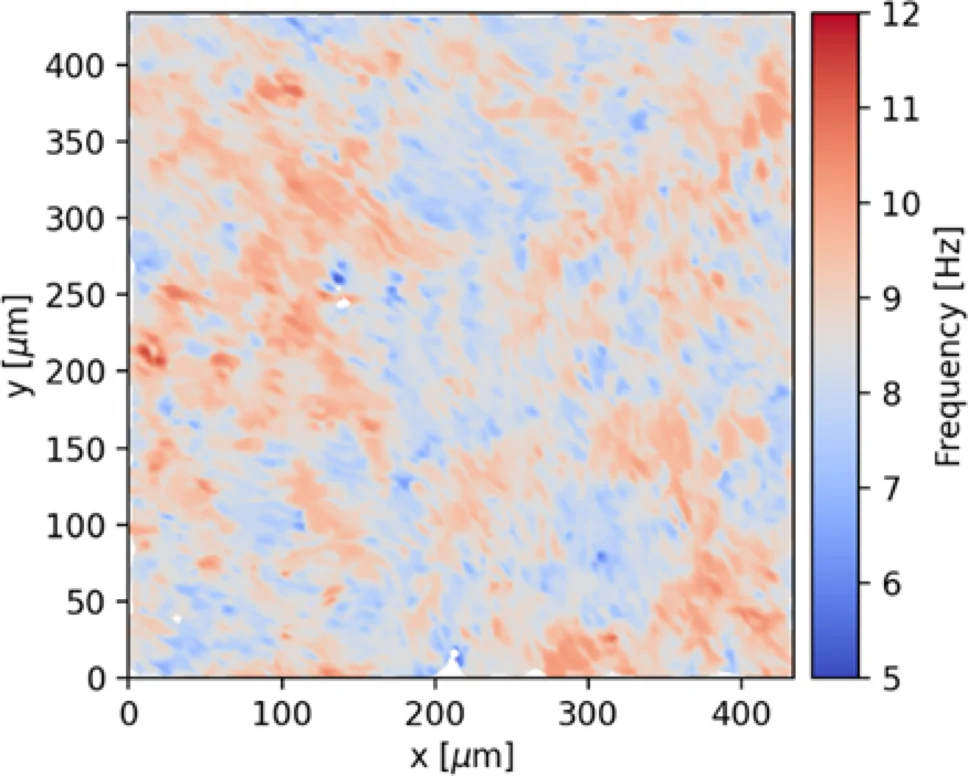
Typical spatial distribution of the CBF referred to as the activity map. White spots indicate missing values, or inactive/invalid pixels

The **active percentage (*α*)** is defined as the percentage of active and/or valid pixels in the activity map (Fig. 6) representing the position-resolved CBF distribution in the entire field of view. It is therefore based on the computation of the activity map. First, a CBF value is assigned to each pixel. Then, for each pixel, we check whether it meets the validity criteria to determine if the frequency indeed results from the movement of the cilia. To do this, we not only consider a pixel’s intensity fluctuations but also the fluctuations of the local optical flow. From an intuitive perspective, we could say that we are also checking whether the intensity fluctuation is indeed caused by a planar movement within the sample plane. The algorithms determining the activity map are rather complex and are described in the Online Supplement (Sect.  2.2).

#### Frequency correlation (${\boldsymbol{\xi}}_{\boldsymbol{\nu}}$)

The spatial distribution of the CBF is not completely random (Fig. 6) but exhibits a certain degree of local correlation, with a distinct patchy structure. To quantify the average size of these patches, we make use of the recently introduced **frequency correlation length** ${\boldsymbol{\xi}}_{\boldsymbol{\nu}}$ (Schneiter et al. 2023).

**Fig. 7:**
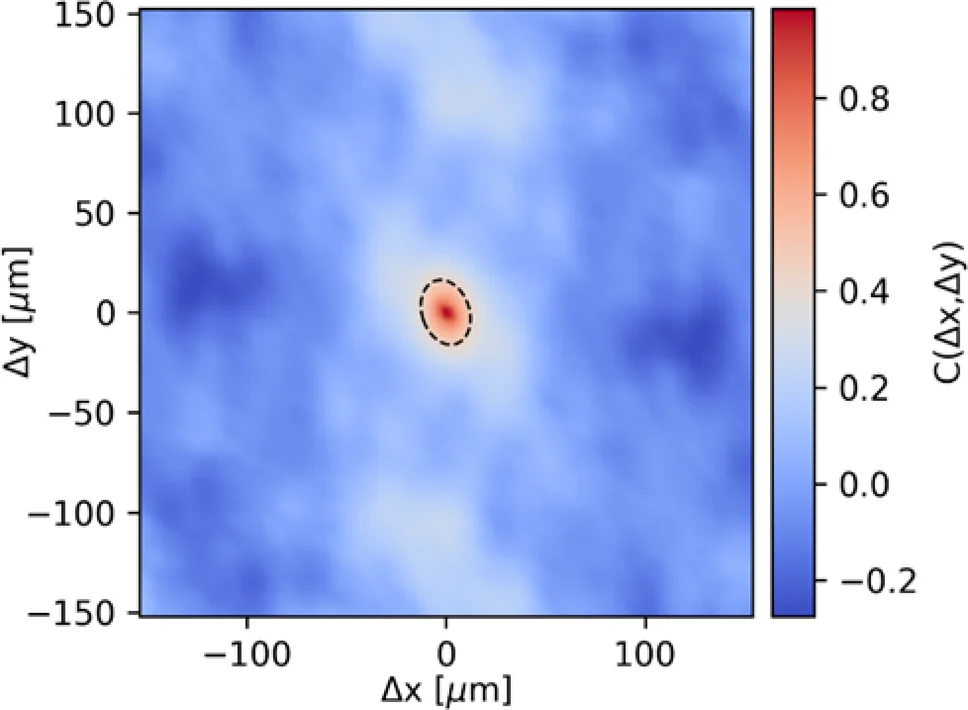
Two-dimensional spatial autocorrelation of the activity map shown in Fig. 6. It features an ellipsoidal peak whose size is measured by ${\xi}_{\nu}^{2}$, where ${\xi}_{\nu}$ represents the frequency correlation length

The frequency correlation can be understood as the average distance over which the CBF roughly remains preserved. Another interpretation concerns its square (${\xi}_{\nu}^{2}$), which can be seen as the average patch size over which the frequency roughly remains preserved. It is important to note that, strictly speaking, ${\xi}_{\nu}^{2}$ does not directly represent the actual patch size, but is instead correlated with it. The two-dimensional autocorrelation function of the activity map is computed from the activity map’s spatial power spectrum by exploiting the Wiener-Khinchin theorem (Oppenheim 2009). Please note that the computed spatial autocorrelation of the activity map is free from wrap-around artifacts (thanks to zero-padding at the boundaries) and uses an unbiased correction for missing values (inactive pixels in the activity map). See Online Supplement Sect.  2.3. for a more detailed description. Figure 7 shows the autocorrelation function of the activity map shown in Fig. 6. In the autocorrelation function, we identify all pixels surrounding the central global correlation peak that exhibit a correlation greater than ${e}^{-1}$; the resulting area is then defined as ${\xi}_{\nu}^{2}$.

#### Filtering in the time domain to highlight mucociliary activity

Before the collective motion pattern of cilia, as represented by the metachronal wave field, can be analyzed, the image sequence must be filtered in the time domain. The filtering essentially removes the static background as well as high-frequency noise, which highlights the dynamics of actual interest. This is achieved by applying a pixel-wise band-pass filter in the temporal frequency domain, which removes all frequencies below the lowest frequency still considered part of the fundamental CBF-peak and all frequencies above those corresponding to the 7th harmonics. Video S2 and Video S4 show the stabilized and temporally filtered versions of Video S1 and Video S3, respectively (see Online Supplement).

#### Analysis of spatial correlations within the metachronal wave field

**Fig. 8:**
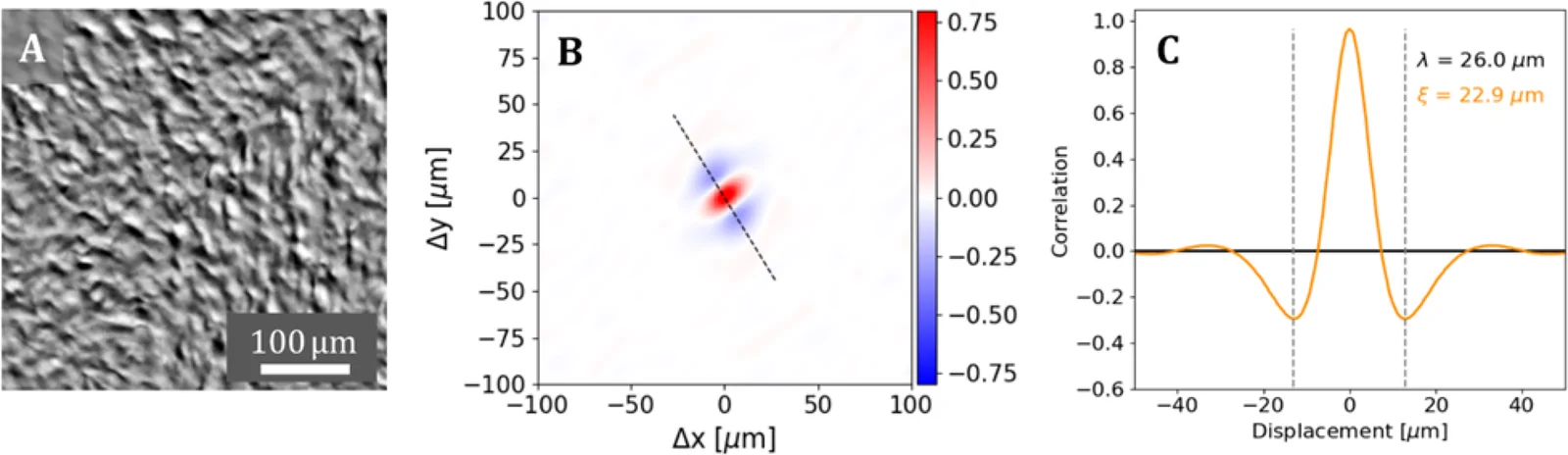
**(A) **Single frame extracted from a preprocessed high-speed video showing the dynamic surface modulations of the mucus surface. Wave-like patterns are evident, characterized by short, transient wavelets that gradually dissipate in both space and time. **(B)** The colormap displays the average spatial autocorrelation (averaged over all video frames). **(C) **Line profile across the distinct central wave structure clearly visible in the averaged spatial autocorrelogram shown in panel B

To analyze the mean spatial structure of the wave-like field, the mean two-dimensional spatial autocorrelogram over all individual video frames is computed. Like the spatial autocorrelation of the activity map, the spatial autocorrelation of each frame in the dynamically filtered image series (Fig. 8A) is computed using zero-padding at the margins to avoid wrap-around artifacts and inactive pixels are excluded from the analysis. An example of a mean two-dimensional spatial autocorrelation is shown in Fig. 8B. As can be seen, the mean autocorrelogram shows a wave-like structure at the center, which can be interpreted as the average wave-structure. A central maximum and the first minima are clearly pronounced around the center of the autocorrelogram. With increasing distance from the central correlation peak, the correlation decreases markedly. This indicates that the local spatial correlation – and thus the collective motion pattern of the cilia – is only strongly coordinated over a distance of about one wavelength.

The black dashed line shown in in Fig. 8B is automatically fitted through the central maximum and its adjacent minima. Figure 8C displays the corresponding correlation profile. As indicated by the two grey vertical dashed lines, the distance between the minima is defined as the **wavelength (**$\boldsymbol{\lambda}$**)**. In addition to the wavelength, we defined three further parameters characterizing the local spatial correlation of the collective motion pattern of cilia: (1) **one-dimensional spatial correlation length (**${\boldsymbol{\xi}}_{1\boldsymbol{D}}$**)**, which is derived from the profile shown in Fig. 8C and reflects the decay rate of the absolute correlation, which is approximately exponential. (2) The **two-dimensional spatial correlation length** ${(\boldsymbol{\upxi}}_{2\boldsymbol{D}}$**)** is determined analogously by the frequency correlation length – meaning that the same approach was used, but for the mean local spatial autocorrelogram. Finally, we also assess the elongation of the central correlation peak in the mean local autocorrelogram by fitting an ellipse. The ratio of the major to the minor axis of the ellipse fitting the central peak in Fig. 8B is referred to as the **local anisotropy ratio (**$\boldsymbol{\zeta}$**)** in the following. Typically, the elliptical peak is elongated perpendicular to the wave propagation direction, indicating stronger lateral coherence, and compressed along the direction of propagation, reflecting the wave periodicity. *ζ* reflects how directionally organized the collective ciliary motion is – higher values indicate stronger alignment and coherence, while lower values point to spatial disorder or isotropy.

#### Space-time correlations – Wave speed and wave disorder

To measure the average propagation speed of the wavelets and the spread in their propagation direction, the preprocessed video is subdivided into square subvideos, each having a side length of about three times the spatial correlation length (${\xi}_{1D}$). To determine the mean wavelet speed, the cross-correlation peak in the space-time correlogram of each subvideo is tracked. This allows assigning a local average wave propagation velocity (speed and direction) to each subvideo. The **wave propagation speed (v**_**w**_), represents the average wave speed taken over all subvideos. See Online Supplement (Sect.  2.4) for a more detailed explanation. The directions of wave propagation velocities are used to determine the **wave disorder** $\boldsymbol{\gamma}$, which is defined as ${\upgamma}=1-{R}^{2}$ and simply corresponds to the directional, or circular, variance (Mardia 2000).

#### Mucociliary transport speed

To determine the **mucociliary transport speed v**_**T**_, we followed the same procedure as described previously (Schneiter et al. 2023). In summary, a small amount of lightweight, dry puffball spores (from Calvatia excipuliformis) was blown onto the mucus surface. The particle tracking was performed with the Cilialyzer software and was based on a local extremum tracker within a manually adjustable window size. The resulting noisy particle traces were first slightly smoothed and then fitted with a univariate spline, whose curve length was subsequently used to determine the mucociliary transport speed. This procedure is highly robust and capable of handling arbitrary particle trajectories.

## Results

### Comparison between reflected- and transmitted-light microscopy

**Fig. 9:**
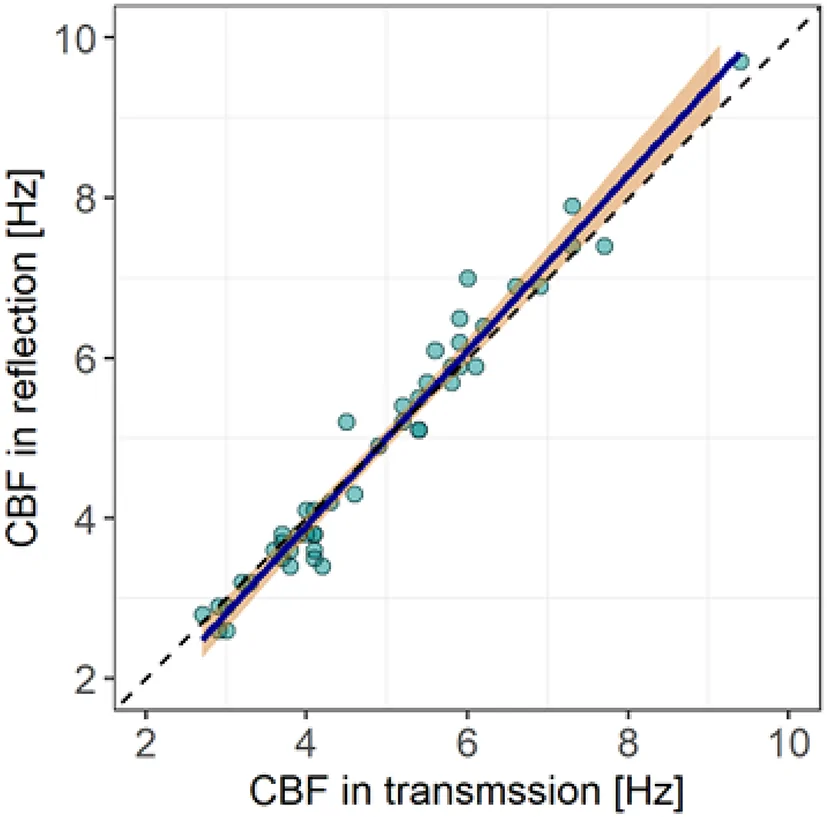
Comparison between CBF-values as determined from two videos, one taken in transmission and one taken in reflection for the same spot on the ALI culture. Measurements were performed sequentially, with a time delay of approximately 2 min between the two modes. Data was acquired from 12 inserts derived from 12 different donors. Dashed line: identity function. Continuous blue line: linear model fit. Region shaded in orange: 99% confidence interval

To ensure that the dynamic modulation of the mucus surface indeed reflects the collectively coordinated motion of the underlying cilia, we used the experimental setup shown in Fig. 3 to compare the collective motion of cilia as imaged in reflection and transmission at the same location. It is important to note that the field of view (FOV) used to determine the CBF in reflection was approximately four times larger than the FOV in transmission. However, careful alignment of the centers of the fields of view was ensured for each acquisition. Figure 9 shows a comparison between the CBF values obtained from transmission and reflection imaging, revealing an excellent agreement between the two methods. To introduce variability of the CBF, cell culture plates were taken from the incubator and directly examined at room temperature (meaning that measurements were performed while cultures gradually equilibrated to room temperature).

**Fig. 10:**
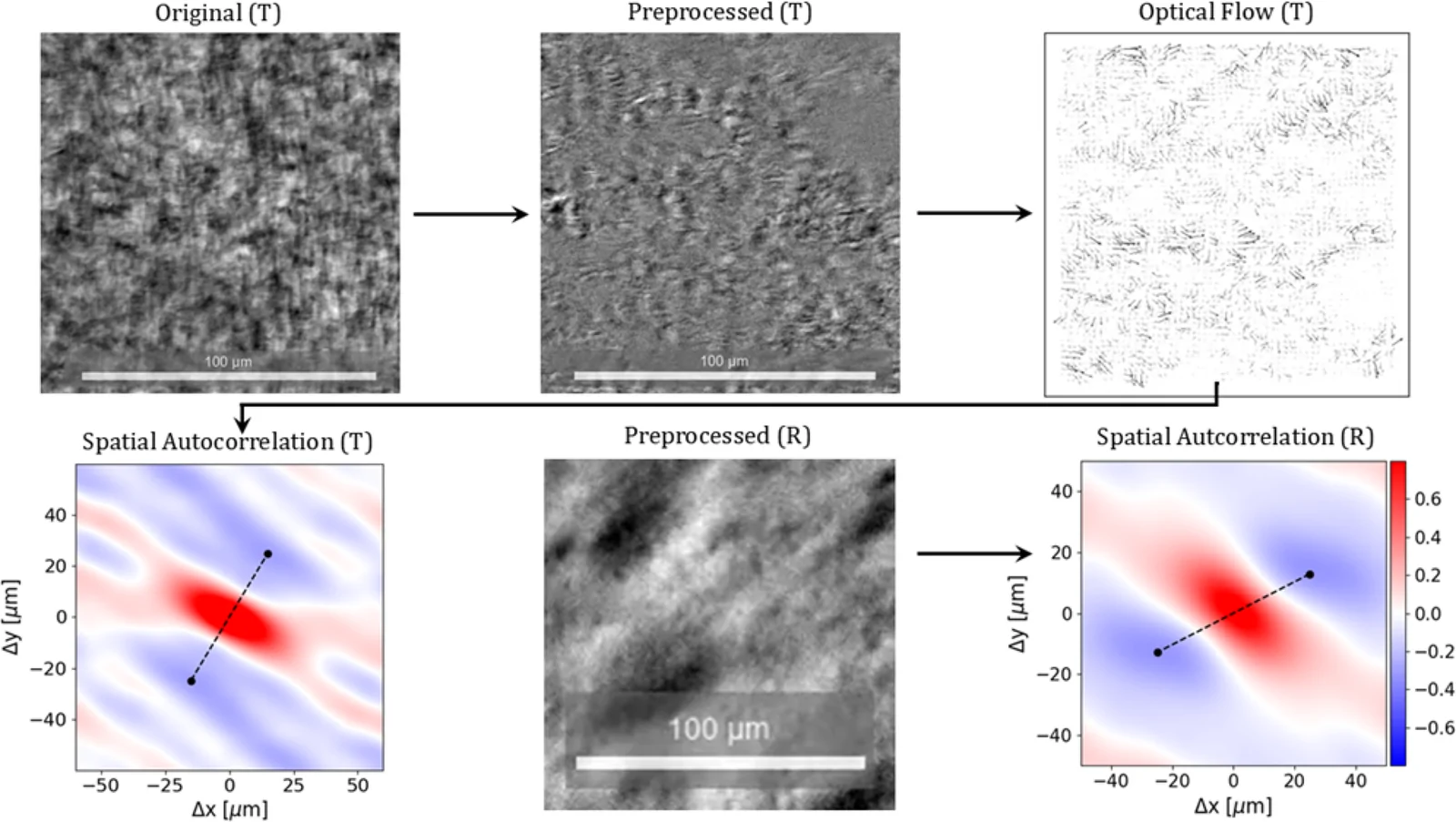
Comparison of Transmission and Reflection recorded at exactly the same sample location. From top left to bottom right: The original recording was stabilised and temporally filtered. Based on this preprocessed version, the dense optical flow was computed (shown here in a down sampled form). Finally, at the bottom left, the mean autocorrelation of the optical flow is shown, from which the wavelength was determined (indicated). The recording in reflection and the mean spatial autocorrelation correspond to the same location. An animated version of this illustration can be found in the supplementary material

Acquiring recordings with transmission microscopy that allow to determine the metachronal wavelength is challenging. Therefore, we demonstrate the agreement between the in-situ wavelength as inferred from reflection and transmission measurements with a representative example. In contrast to the recordings obtained by reflection microscopy, the transmission recordings do not directly show the collective beating pattern of the cilia, but rather a snapshot of their projected displacement. For this reason, the wavelength cannot be directly determined from the autocorrelation of the frames after time domain filtering. In transmission recordings, the metachronal wavelength was thus determined as follows (see Fig. 10; note that Video S5 in the supplementary material represents an animated version of this Figure): after preprocessing (mainly stabilization and temporal filtering), the dense optical flow was computed using the algorithm proposed by Farnebäck (Farnebäck 2003), which estimates the motion of the cilia between two consecutive frames based on a local polynomial expansion. The resulting velocity field was then autocorrelated separately in its *x*- and *y*-components, and the resulting two-dimensional spatial autocorrelations were subsequently averaged. This was done for each optical flow frame yielding the average spatial autocorrelation. In this, as well as in several other examples where we were able to determine the wavelength in both transmission and reflection, the wavelength agrees exactly in both recording modes (in the example shown in Fig. 10, the wavelength amounts to 57.8 ${\upmu}\mathrm{m}$) – which is compelling given the different underlying imaging as well as computational approaches.

### Qualitative visual observations of mucociliary phenomena

**Fig. 11:**
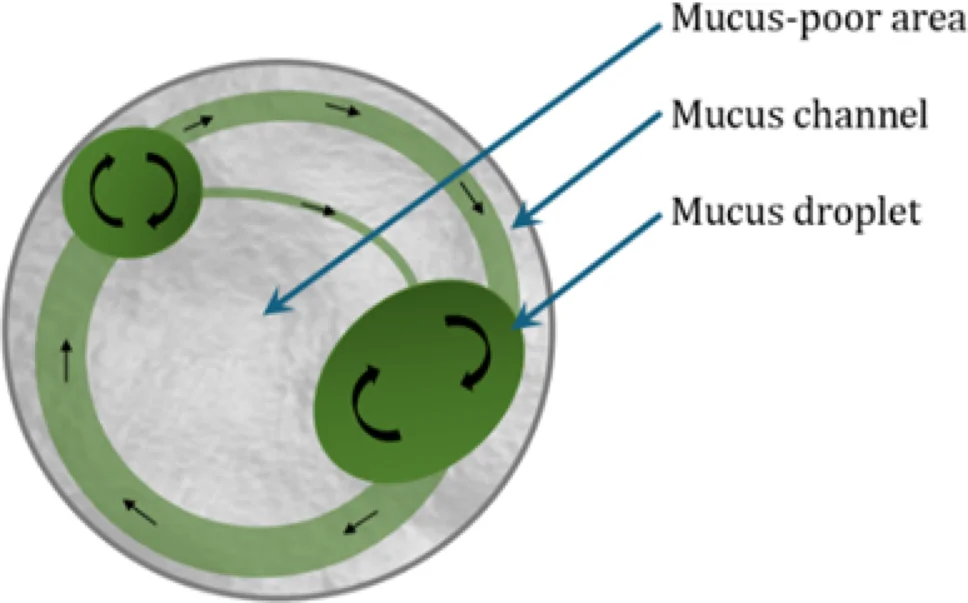
Typically, the observer encounters one of the following mucus-related situations in respiratory ALI cell cultures: (**1**) absence of a visible mucus layer and no mucociliary transport; (**2**) mucus transport restricted to a narrow path or channel; or (**3**) formation of a thick mucus droplet that often rotates around its centre

When visually observing mucociliary activity, it quickly becomes evident that the mucus is not evenly distributed across the cell culture and that it is the amount of mucus present in a specific field of view, which – together with its position within the culture (central or peripheral) – mainly determines the phenomenological character of the observable mucociliary activity. Based on numerous observations of the mucociliary activity in healthy cultures, clear patterns emerge with typical manifestations of the phenomena that can be categorized as follows (see also Fig. 11): (1) mucus-poor areas: hardly any mucus is present in these areas. Consequently, no transport or only extremely slow transport ($\lesssim1\frac{{\upmu}\mathrm{m}}{\mathrm{s}}$) can be observed in these locations. Due to the low mucus layer height, the collective dynamics of cilia are particularly well visible (due to the shallow mucus layer, the mucus surface is modulated more strongly by the underlying cilia resulting in better contrast). (2) Mucus channels (see also Video S6): mucus is transported along channels of different widths. The collective dynamics of cilia are still clearly observable. Due to the relatively thick mucus layer, however, the ciliary dynamics appear somewhat blurred and not as clearly as in mucus-poor areas. (3) Mucus droplets (see Video S7 for example): larger amounts of mucus seem to coalesce into roughly circular droplets. Mucociliary transport is expressed by the rotation of the mucus droplet, as has been observed previously. On sites with thick mucus droplets, the dynamics of the underlying cilia can no longer be reliably detected (as the modulations of the mucus surface are too small to be detected).

Along the circular walls of the inserts, a mucus channel is typically present and mucus gets transported along the insert’s walls. Moreover, as previously observed, the wave propagation directions tend to be more disordered in the center of the cultures compared to the periphery, most likely due to a less well-aligned physical orientation of the cilia.

In summary, the typical pattern an observer encounters consists of a few rotating mucus droplets connected by mucus channels of different heights and widths and in-between areas showing hardly any mucus. We presume that these three characteristic transport patterns constitute a dynamic conveyor belt where channels can be considered as inflows and outflows of the droplets. As a stationary situation seems unlikely, we suspect that the droplet-channel structure changes over time (and thus cause the droplets to move – not least due to the continuous mucus secretion).

Finally, we would like to emphasize that a correlation between the metachronal wavelength of the collective motion of cilia and the height of the mucus layer can be observed clearly.

### Quantitative characterization of mucociliary activity in healthy ALI cell cultures

**Fig. 12:**
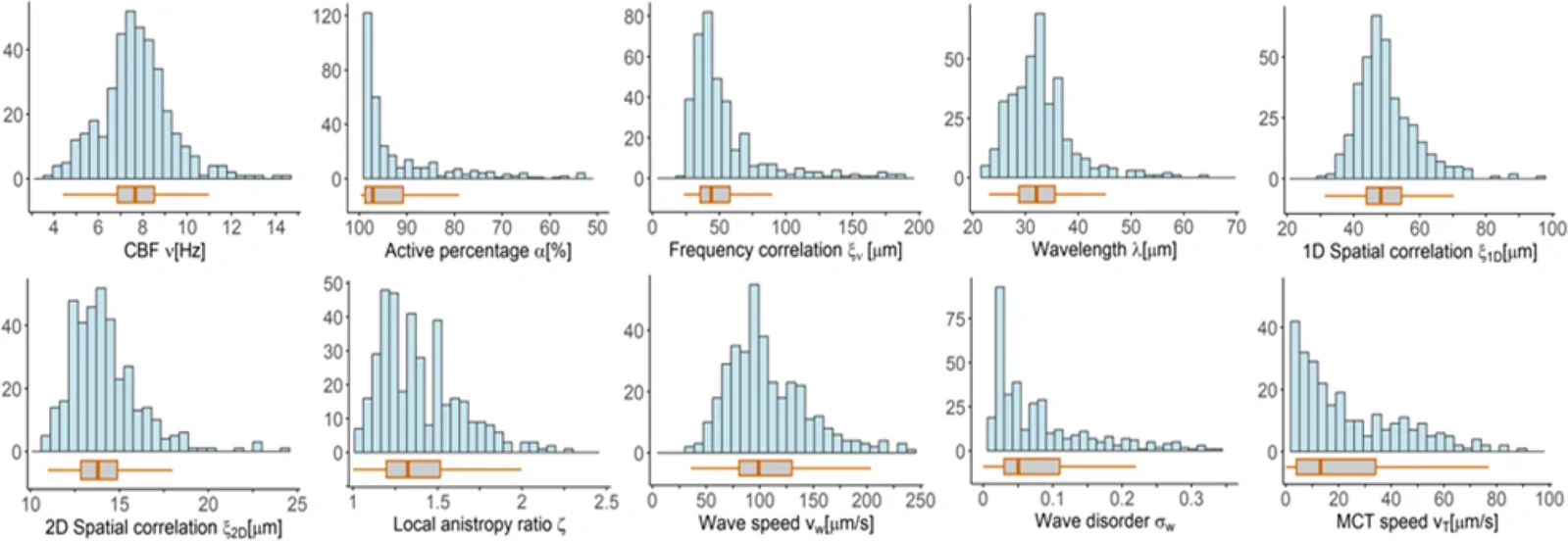
Distribution of the determined values for the set of ten observables characterizing mucociliary activity, illustrated by histograms and boxplots. Boxes represent the interquartile range (IQR), the central line indicates the median, and whiskers extend to the most extreme data points within 1.5 times the IQR. The values were determined from 65 cell cultures derived from 20 healthy individuals (1 to 5 inserts/donor), for each cell culture 3 to 9 videos were evaluated. The recordings were done using the research system. Measurements of transport speed were based on recordings taken from regions with a substantial amount of mucus with the mucus transport settings, while all other observables were obtained from mucus-poor regions with the mucociliary dynamics settings

As mentioned in Sect.  2.2.1, two types of recordings were performed at different locations with different camera settings: mucociliary dynamics and mucociliary transport. Recordings of mucociliary dynamics were consistently performed in areas with little or no mucus. On the other hand, mucociliary transport obviously requires the presence of mucus that can be transported. Accordingly, very low transport values correspond to cultures that showed only little mucus. Figure 12 shows the distribution of values for the set of 10 observables in healthy cell cultures.

While active percentage, wave disorder, and mucociliary transport speed display clearly skewed distributions, the observables describing the collective dynamics – such as CBF, frequency correlation, wavelength, spatial correlation lengths, local anisotropy ratio, and wave speed – exhibit mostly symmetric and unimodal distributions, often approximating a normal distribution or showing only moderate skewness. The latter observables are therefore consistently distributed across cultures.

#### Most striking mutual correlations among observables

In the following, the most striking mutual correlations among the measured observables are presented. To mitigate the potentially distorting effect of outliers, the correlation analysis was restricted to data within the range given by the boxplots’ whiskers.

##### Patchwork character of the collective mucociliary dynamics

As has been reported previously (e.g. see (Ryser et al. 2007)), the collective coordination of cilia exhibits a typical patchwork character: the coordination of cilia is restricted in both space and time and typically exhibits areas of collective coordination expressed by wavelets with a certain degree of disorder causing the correlation to fade in space and time.

**Fig. 13:**
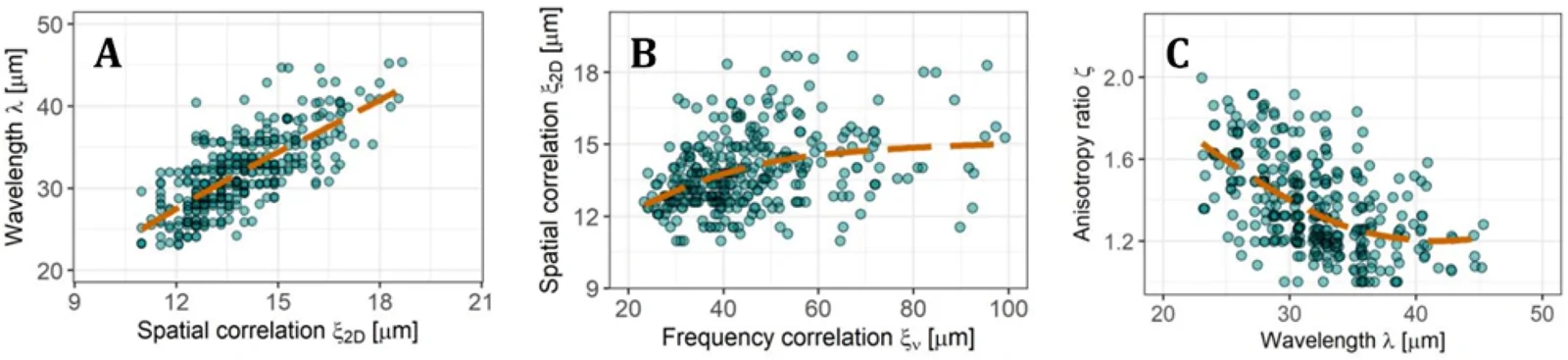
The scatter plots illustrate three mutual associations among the observed parameters values; each dot corresponds to one high-speed video. The dashed dark orange line represents a LOESS non-parametric regression and serves to indicate the underlying trend. (**A**) Pronounced correlation between the metachronal wavelength and the spatial correlation. (**B**) The data shows a weak association between the spatial correlation and the frequency correlation. (**C**) On average, there is a relatively clear (negative) correlation between the anisotropy ratio and the wavelength

As can be seen in Fig. 13A, it is evident that the metachronal wavelength is strongly associated with the spatial autocorrelation length: longer wavelengths are strongly linked to collective beating exhibiting extended spatial correlations. Moreover, the collected data shows that the spatial correlation decays within the distance of a single wavelength.

Both the activity map and the collective beating pattern exhibit a patchwork-like structure (Figs. 6 and 8A). This naturally raises the question of whether the characteristic patches observed in the activity map correlate with the spatial confinement in the collective beating pattern, and whether the latter might, to some degree, be shaped by local discontinuities in the CBF. The scatterplot in Fig. 13B, in which the spatial correlation length is plotted versus the frequency correlation length, provides insight into this important question. As can be seen, the data indicates that variations in spatial coordination are only weakly related to variations of the frequency correlation length.

##### Wavelength-dependent shift from planar to rotational ciliary motion

Figure 13C shows that the anisotropy ratio is negatively correlated with the wavelength, which can be interpreted as follows. A decrease in the anisotropy ratio can arise from two distinct sources. On the one hand, a highly disordered wave field (i.e. a large value for *γ*) could result in an apparently low local anisotropy, since the autocorrelogram is computed over a large field of view. However, this possibility can be ruled out, as anisotropy is not correlated with the wave disorder *γ* (data not shown). Therefore, the most likely interpretation of the present result is that ciliary beating becomes more rotational with increasing metachronal wavelength – which was observed, and which also explains why coupling perpendicular to the wave propagation direction increases with the wavelength. As will be discussed, longer wavelengths are highly likely related to a thicker mucus layer thickness.

### Identification of confirmed PCD cases based on multivariate discriminant analysis

As shown earlier, the measured values for the various physical observables are consistently distributed. This is a highly important finding per se, as smaller variability makes it easier to detect abnormalities – be it for diagnostic applications or for the identification and quantification of changes in drug testing.

The collective mucociliary activity, as imaged by reflection microscopy, resembles a superposition of wavelets fading in space and time. Consequently, the mean CBF and the mean wavelength are the two most fundamental characteristics to describe the collective ciliary activity (i.e. the temporal and spatial periodicity of the collective beating pattern).

**Fig. 14:**
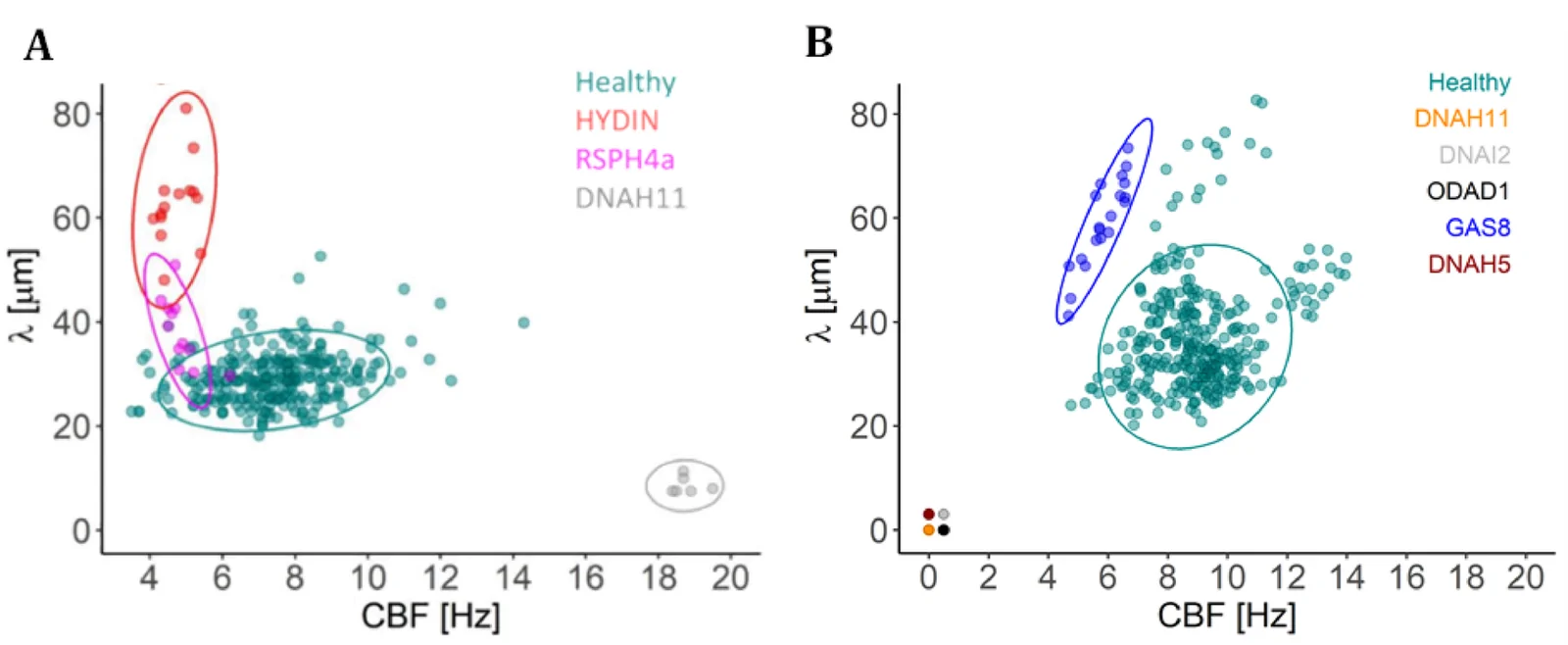
**A**) Data collected using the research system and 65 inserts of 20 healthy donors. (**B**) Data collected using the clinical system. Each data point represents a video for which we simultaneously determined the average CBF and the average metachronal wavelength. One can see that the data shows a high consistency, which is further highlighted by the data ellipses indicating a confidence level of 95% assuming a bivariate t-distribution. Please note that the data would spread much less strongly, if averaging within each individual would be applied

As these two parameters have found to be only weakly correlated, we use them to illustrate the concept of multivariate discriminant analysis to identify PCD in the following. The two panels shown in Fig. 14 illustrate the concept of multivariate discriminant analysis in two dimensions. The data shown on the left (A) was generated by using the research setup, whereas the data shown on the right (B) was generated by using the clinical system. The panels show the distribution of simultaneously measured values for ciliary beat frequency (CBF) and metachronal wavelength in airway ALI cultures obtained from healthy volunteers and from PCD patients with genetically confirmed diagnoses. Each point in the scatter plot represents a single video recording. The ellipses indicate a two-dimensional 95% confidence region for the dataset in each category (e.g. on the left: healthy, HYDIN (usually manifests with reduced bending and beating amplitude), RSPH4a (showing predominantly rotational beating pattern), DNAH11 (typically hyperfrequent beating with reduced bending amplitude and stiff ciliary motion, often predominately immotile in cell cultures), corresponding to the area that would encompass 95% of the data points under the assumption of a bivariate t-distribution. Please note that the ellipses are only a visual tool; however, they clearly illustrate that the values for the different affected genes are well separated, and that incorporating the wavelength provides substantial additional discriminatory power. Please note, on the right side, no collective activity could be measured in DNAH11, DNAI2 (mostly immotile cilia), ODAD1 (mostly immotile cilia, some with pathologic residual movement), and DNAH5 (predominantly totally immotile cilia in cell culture with occasional slow residual uncoordinated movement). Recordings were nevertheless performed; however, these show only noise. Therefore, the CBF and the wavelength for these data were set to 0 (in the figure, the points were manually shifted so that all colors can be distinguished).

## Discussion

### Coupling between ciliary beating and the overlying mucus layer surface

With our multimodal setup, we imaged ciliary motion in transmission at the same location as the modulation of the mucus surface and compared the resulting values for the CBF and the metachronal wavelength between both imaging modalities. As shown in Sect.  3.1, the CBF and the metachronal wavelength agree very well between recordings taken in transmission and reflection. The observed deviations between the two imaging modalities are most likely due to two main sources of variation: the size of the field of view does not match exactly, and there is a short time delay between the two recordings taken from the same location.

We conclude that our results unequivocally show that dynamic modulations of the mucus surface are indeed linearly related to the underlying motion of cilia and thus the motion of the mucus surface adequately reflects the underlying activity of cilia. From a physical point of view this is not surprising, since anything else other than a linear coupling between the mucus surface modulations and the underlying collectively coordinated ciliary activity is hard to imagine when considering the low Reynolds number of the involved fluid mechanics.

### Patchwork character of mucociliary activity

Previous studies have shown that the collective motion of cilia exhibits a typical patchwork character, meaning that the collective coordination can be characterized in terms of wavelets fading in space and time. This naturally raises the long-standing question about the origin of the patchwork-like coordination mechanism. The observations made in this study contribute to answering this important question. As shown in Sect.  3.3.1, there is only a weak correlation between frequency correlation length and the spatial correlation length, suggesting that the local spatial decorrelation is unlikely to result from frequency discontinuities, which has been presumed previously. Our results suggest that, on the one hand, the decorrelation of coordination is not primarily caused by differences in CBF but rather by other factors such as phase differences, variations in beat direction, differences in the CBP of individual cilia and the fluid-mechanical coupling caused by spatial variations of the mucus height or mucus rheology. We observed that the spatial correlation at a given location varies over time (in particular when thinner or thicker mucus passes over it). Therefore, the patch-work character cannot be attributed solely to the underlying physical cellular structure, such as the distribution of ciliated and unciliated cells, which we presumed previously (Schneiter et al. 2020). Information of the frequency and its spatial distribution alone are therefore not sufficient to describe the coordination of collective ciliary motion, which highlights the importance of wavelength and spatial correlation length for diagnostic or drug testing purposes and can only be measured reliably with reflection microscopy.

### Wavelength-dependent shift from planar to rotational ciliary motion

Figure 13 shows that the anisotropy ratio is negatively correlated with the metachronal wavelength. This relationship can be interpreted as follows. Strictly speaking, a decrease in anisotropy could arise in two different ways. First, a strongly disordered wave field (large γ) could lead to a reduced anisotropy, since the autocorrelogram is computed over a large field of view. However, we can exclude this explanation because anisotropy does not correlate with wave disorder. The present finding is therefore most likely interpreted as evidence that the ciliary motion pattern becomes more rotational with a longer wavelength, whereas it tends to be more planar in regions with a shorter wavelength.

Moreover, we observed a clear positive correlation between metachronal wavelength and mucus layer height (quantitative measurements based on OCT measurements will be reported in detail elsewhere (manuscript in preparation)). Overall, our quantitative analyses are consistent with our qualitative observations – based on reflection and transmission imaging – that an increase in mucus thickness is associated with a transition from predominantly planar to more rotational beating of individual cilia (oscillating within a dense array of cilia, all exhibiting a similar motion pattern), which is reflected at the collective level by longer metachronal wavelengths. The height and rheology of the mucus layer may influence the rotational aspects of ciliary motion and may also be one of the reasons why some of the RSPH4a data points in Fig. 14A overlap with those of healthy controls.

We hypothesize that in a very thin mucus film, the polymeric network may behave elastically, imposing restoring forces that restrict the beat amplitude of individual cilia. This mechanical constraint might favor a more planar beat pattern and reduce hydrodynamic coupling between neighboring cilia, which manifests as shorter metachronal wavelengths. In contrast, in a thicker and predominantly viscous mucus layer, cilia can adopt more three-dimensional, rotational trajectories enabling stronger hydrodynamic interactions and longer metachronal wavelengths.

### Advantages of computational high-speed video reflection microscopy

Compared to transmission microscopy, reflection microscopy offers a number of advantages: (1) Quantifying the collective ciliary dynamics via transmission microscopy requires individual cilia to be optically resolved well enough to allow the tracking of their motion. This typically requires a cutting-edge, high-cost microscope setup. In contrast, we demonstrate here that a standard upright microscope is sufficient to clearly visualize the collective motion of cilia in reflection. Since we focus on quantifying the collective dynamics, resolving individual cilia is not necessary. (2) Bigger FOV: Because individual cilia do not need to be resolved, much larger and more representative FOV can be imaged at once. This is not feasible with transmission microscopy, which requires a high numerical aperture – and therefore a high magnification (resulting in small fields of view) – to achieve sufficient resolution of individual cilia. (3) Determining the wavelength from transmission recordings is only possible if the image quality is very high. This is clearly not feasible in a clinical setting, particularly due to the need for high-throughput processing.

### Biased FOV-selection and potential of CVRM in diagnosing PCD

As mentioned earlier, we recorded mucociliary transport and collective mucociliary activity at different locations on the sample. For the collective activity, we prioritized FOV for which the collective beating pattern of the cilia could be captured with maximum contrast and resolution. This was achieved when the mucus layer was relatively thin, as cilia-induced modulations of the mucus are more pronounced under these conditions, resulting in a particularly high-contrast representation of the metachronal beat pattern. On the other hand, we qualitatively observed early on that the metachronal wavelength correlates with the height of the mucus layer. It is important to emphasize that this is not an artifact of reflection microscopy, but rather a genuine observation: cilia tend to exhibit longer wavelengths and a more rotational beat pattern beneath thicker mucus layers. For this reason, we initially focused on selecting FOVs with thinner mucus layers – with the intention of minimizing the substantial variability in mucus layer height to facilitate the detection of deviations in collective dynamics, particularly in the context of PCD diagnostics. In retrospect, it would be interesting to sample random positions instead, as correlations between parameters would likely appear even stronger if we had not constrained their variance through the selective FOV choice. For transport measurements, in contrast, we deliberately chose regions with a considerably thicker mucus layer, as areas with minimal mucus height exhibit little to no measurable transport. Retrospectively, it would be valuable to acquire data from randomly selected locations and simultaneously record transport; ideally, this would even include in situ measurements of the mucus layer height. Considering the correlations and variances – i.e. the covariance structure of multivariate healthy reference data – methods such as the Mahalanobis distance (Mahalanobis 1936) can then be used to classify new patient data as normal or abnormal. Soon, an updated version of Cilialyzer will be released, which will enable simultaneous quantification of transport speed and the parameters characterizing collective mucociliary motion.

Regarding particle tracking, we would like to note that adding suspended particles to ALI cultures temporarily slightly alters mucus rheology. Additionally, in reflection microscopy, applying an aqueous solution to the apical surface temporarily reduces mucus reflectivity – both because of the added fluid layer and because dilution decreases the mucus’ backscattering capacity.

Figure 14 highlights the potential of CVRM for PCD diagnostics and demonstrates that with a commercially available, cost-efficient system, all of the PCD cases with known genetics that we tested were reliably detected. However, the careful reader will notice differences between the left and right panels for healthy controls and DNAH11. This does not mean that the systems produced different values for the same samples as the samples were not identical in the first place. Rather, this is due to the fact that in the research setup the temperature was set slightly lower (about 34 °C instead of 37 °C), resulting in somewhat lower CBF values in healthy volunteers. In addition, on the left greater care was taken to record at locations with minimal mucus and high contrast, which explains why the wavelengths in healthy volunteers were found to be lower on the left, and why in DNAH11 it was in some cases possible to capture mucociliary activity in certain regions. Overall, we would like to emphasize once more that the differences between normal and PCD would become even more pronounced after subject-specific averaging.

## Conclusion

We showed here that reflection compared to transmission video microscopy has important advantages that allow the quantitative analysis of the collective ciliary activity in primary air-liquid interface cell culture. This allows to distinguish between cell cultures from healthy controls and people with PCD, what could be further development for the diagnosis of respiratory diseases.

## Supplementary Information

Below is the link to the electronic supplementary material.

Supplementary Material 1

Supplementary Material 2

Supplementary Material 3

Supplementary Material 4

Supplementary Material 5

Supplementary Material 6

Supplementary Material 7

Supplementary Material 8

## Data Availability

Data generated for this publication can be obtained upon resonable request from the corresponding author.
