# Supplementary material for: Quantitative analysis of mucociliary activity by computational high-speed video reflection microscopy
Source: Eur Biophys J. 2026 Mar 16;55(4):663–77. doi: 10.1007/s00249-026-01835-6 (PMC13672628; doi:10.1007/s00249-026-01835-6)
Supplement: Supplementary file 1 [file 249_2026_1835_MOESM1_ESM.docx]

**Online Data Supplement of**

**Quantitative analysis of mucociliary activity by computational high-speed video reflection microscopy**

Martin Schneiter ^1,2^, Julian Wyss ^2^, Jeremy Schori ^1,3^, Patrik Arnold ^3^, Martin Frenz ^1^, Pietro Cicuta ^4^, Stefan A. Tschanz ^2^, André Stefanov ^1^ and Loretta Müller ^5,6^

^1^ Institute of Applied Physics, University of Bern, Bern, Switzerland

^2^ Institute of Anatomy, University of Bern, Bern, Switzerland

^3^ Bern University of Applied Sciences, Engineering and Information Technology, Biel, Switzerland

^4^ Cavendish Laboratory, University of Cambridge, Cambridge, United Kingdom

^5^ Division of Paediatric Respiratory Medicine and Allergology, Department of Paediatrics, Inselspital, Bern University Hospital, University of Bern, Bern, Switzerland

^6^ Lung Precision Medicine (LPM), Department of BioMedical Research (DBMR), University of Bern, Bern, Switzerland

1. Temporary increase in CBF and metachronal wavelength following cell wash

The mucociliary activity was recorded in 11 ALI inserts obtained from 11 healthy donors over 48h after washing the cells. For each time point and insert, 10 videos were recorded in reflection-mode. CBF and wavelength decrease within the first 24 hours after washing (Figure S1). While CBF stays stable until 48 hours, the wavelength increases again. We hypothesize that the metachronal wavelength gradually increases again after 24 hours due to the reformation of the mucus layer. Those results were the basis to choose the timepoint of 24 hours after washing for most of the recordings.

Figure S1: Temporal evolution of CBF and metachronal wavelength measured at non-equidistant time points after cell washing. The black line indicates the median and the grey-shaded area represents the interquartile range.

2. Cilialyzer-Algorithms used to compute the observable values

Please note that the algorithms to determine the observables partly resemble those which have previously been presented in Schneiter et al, 2023 {Schneiter, 2023 #56}. However, they are partly restated here with several subtle improvements. Please note that only the key computational steps are presented in the following, in order to keep the descriptions as concise as possible.

2.1. Determination of the Ciliary Beating Frequency (CBF)

The ciliary beating frequency (CBF) is determined by averaging the relative power spectral densities (PSDs) corresponding to the temporal intensity fluctuations of pixels within the selected region of interest or the entire field of view (FOV).

The image sequence (or video) can be considered as a three-dimensional array containing the intensity fluctuations and can thus be denoted as $I\left( x,y,t \right)$. The mean image can be denoted as $\left\langle I\left( x,y,t \right) \right\rangle_{t}$, where $\left\langle\ldots\right\rangle_{t}$ represents averaging along the time axis. In a first step, we mean-center the image data by subtracting the mean image from the raw image sequence:

$I_{c}\left( x,y,t \right)=I\left( x,y,t \right)-\left\langle I\left( x,y,t \right) \right\rangle_{t}$.

Subsequently, to reduce noise, $I_{c}$ is slightly smoothed by Gaussian filtering, which can be denoted as a convolution with a Gaussian kernel: $I_{c}^{'}=I_{c}*G_{\sigma=1,w=1}$, where $G_{\sigma,w}$ represents a Gaussian kernel with a standard deviation of $\sigma$ (as measured in $\Delta x, \Delta y$ and $\Delta t$, respectively) and a kernel size of $w$ measured in standard deviations. Note that here, the Gaussian filtering is performed along all axes (i.e. $x,y$ and $t$). In the next step, for each pixel ($x,y)$ an FFT is performed along the time-axis, which can be written as a discrete Fourier transform:

| $\tilde{I}_{x,y}\left( f \right)=\sum_{t=0}^{N_{t}-1} I\left( x,y,t \right)\cdot\exp\left( -2\pi ift/N_{t} \right)$ | 7.1 |
| --- | --- |

where $f$ is a discrete array index representing the dimensionless frequency. $N_{t}$ denotes the number of captured frames.

The frequency index $f\in\{0,1,\ldots,N_{t}-1\}$ corresponds to both, positive and negative physical frequencies. The conversion to physical frequencies (in [Hz]) is given by (assuming an even $N_{t}$):

$$F\left( f \right)=\left\{ \begin{aligned} \frac{f}{N_{t}}\cdot FPS, \mathrm{for} f<N_{t}/2 \\ \left( \frac{f-N_{t}}{N_{t}} \right)\cdot FPS, \mathrm{for} f\geq N_{t}/2 \end{aligned} \right.$$

where $FPS$ denotes the number of frames per second captured by the camera.

The pixel-wise power spectra $P_{x,y}\left( f \right)$ are given by the pixel-wise squared Fourier amplitudes $\left| \tilde{I}_{x,y}\left( f \right) \right|^{2}$ given by Eq. (7.1). Note that only positive frequencies, i.e. all $f$ for which $F\left( f \right)$ > 0, are kept. We can then define the relative (normalized) power spectral density as:

| $\hat{P}_{x,y}\left( f \right)=\left( \sum_{f>0} P_{x,y}\left( f \right) \right)^{-1}\cdot P_{x,y}\left( f \right)$ | 7.2 |
| --- | --- |

Accordingly, the area-averaged relative power spectral density can then be determined according to:

| $\left\langle\hat{P}_{x,y}\left( f \right) \right\rangle_{x,y}=\frac{1}{N_{x}N_{y}}\cdot\sum_{x,y} \hat{P}_{x,y}\left( f \right)$ | 7.3 |
| --- | --- |

To improve the robustness to fit the area-averaged relative PSD, the latter is slightly smoothed with the Gaussian kernel: $G_{\sigma=1,w=2}$ . The resulting function is finally fitted to the fit function $g\left( f \right)$, which is composed of a decaying power function and two Gaussians:

$$g\left( f \right)=a\cdot\left( f+b \right)^{-c}+p_{1}\cdot\exp\left( -\frac{\left( f-\mu_{1} \right)^{2}}{2\sigma_{1}^{2}} \right)+p_{2}\cdot\exp\left( -\frac{\left( f-\mu_{2} \right)^{2}}{2\sigma_{2}^{2}} \right) ,$$

where all the fit parameters are non-negative, i.e. $a, b, c, p_{1},p_{2}, \mu_{1}, \mu_{2}, \sigma_{1}, \sigma_{2}\geq0$.

The two Gaussians are given by $p_{1}, \mu_{1}, \sigma_{1}$ and $p_{2},\mu_{2}, \sigma_{2}$, where these parameters correspond to the peak heights, the mean frequencies and the standard deviations, respectively. Based on these estimates, it is checked whether the second Gaussian peak should be considered as to contribute to the CBF or should be considered as a higher harmonic. Subsequently, the local minima of the fundamental peak determine the CBF-bandwidth, i.e. all frequencies originating from ciliary beating, which we denote as [$f\_1,f\_2]$.

The CBF is finally determined as a weighted average within the CBF-bandwidth given by the two cut-off frequencies, the lower limit $f_{1}$ and the upper limit $f_{2}$. The weight for each frequency is given by the area-averaged relative PSD. The weighted CBF-peak (in [Hz]) and the CBF-peak width represented by the PSD-weighted standard deviation are finally given as:

| $\left\langle F_{CBF} \right\rangle=\left[ \left( \sum_{f_{i}=f_{1}}^{f_{2}} w_{i} \right)^{-1}\sum_{f_{i}=f_{1}}^{f_{2}} w_{i}\cdot f_{i} \right]\cdot\frac{FPS}{N_{t}}\sigma_{F}=\sqrt{\left\langle{F_{CBF}}^{2} \right\rangle-\left\langle F_{CBF} \right\rangle^{2}}$ | 7.4 |
| --- | --- |

Where ${w_{i}=} \left\langle\left〖 \hat{P}_{x,y}\left( f_{i} \right) \right\rangle\right〗_{xy} .$

2.2. Determination of the activity map

The activity map provides information on how the CBF is spatially distributed and can thus be seen as a position-resolved representation of CBF.

In the calculation of the activity map, we first assign a CBF value to each pixel. Then, for each pixel we check whether it meets the validity criteria to determine if the frequency indeed results from the movement of the cilia. To do this, we not only consider the intensity fluctuations of a pixel but also the fluctuations of the local optical flow. From an intuitive perspective, one could therefore say that we are also checking whether the intensity fluctuation is indeed caused by a planar movement within the sample plane. Note that the computation of the activity map, specifically the determination of inactive pixels, has greatly improved since we incorporated the optical flow.

In the following, the individual steps for calculating the activity map are outlined.

In a first step, the image sequence is centered by subtracting the mean image to improve the motion detection performed by Farnebäck’s algorithm as implemented in OpenCV (Open Source Computer Vision Library) {Bradski, 2008 #133}, which determines a dense optical flow field. The resulting optical flow field is slightly smoothed by median filtering (for outlier removal) followed by Gaussian filtering.

In the following, we denote the array representing the activity map as $\mathbf{A}\left[ x,y \right]$, which holds the position-specific CBF for each pixel located at$\left( x,y \right)$ for which $x\in\{1, \ldots,N_{x}\}$ and $y\in\{1, \ldots,N_{y}\}$. Further, the two-dimensional optical flow field can be denoted as s $\mathbf{V}_{\mathbf{x}}\left[ x,y,t \right]$ and $\mathbf{V}_{\mathbf{y}}\left[ x,y,t \right]$ .

The position- or pixel-specific CBF, and therewith $\mathbf{A}\left[ x,y \right]$, is determined according to Eq.7.4 by substituting the average PSD $\left\langle\left〖 \hat{P}_{x,y}\left( f_{i} \right) \right\rangle\right〗_{xy}$ with the pixel-specific PSD.

However, usually only a part of the whole ﬁeld of view displays ciliary activity. To discriminate between pixels corresponding to areas showing ciliary activity, which are referred to as valid pixels and invalid pixels corresponding to areas showing insufficient ciliary activity, we require that valid pixels satisfy multiple conditions. The validity of a pixel located at$\left( x,y \right)$ is finally specified by the binary validity mask $\mathbf{M}_{\mathbf{v}}\left[ x,y \right]$ having the same dimensions as the activity map. A pixel is considered valid, i.e. $\mathbf{M}_{\mathbf{v}}\left[ x,y \right]=1$, if the following conditions are met:

Condition on the optical flow speed: the optical flow speed is given by $\mathbf{S}\left[ x,y,t \right]=\sqrt{V_{x}^{2}+V_{y}^{2}}$. Intuitively, the optical flow speed represents the magnitude of local motion. Therefore, we require that its temporal average $\left\langle\boldsymbol{S}\left[ x,y,t \right] \right\rangle_{t}$ is larger than 10 $\mu$m/s. Further, we determine the temporal variance of the optical flow speed for each pixel. Subsequently, thresholding was performed using Otsu’s algorithm (Otsu, 1979), which selects the variance threshold $\theta_{1}$by maximizing the interclass variance. All pixels (x,y) showing a temporal variance in the flow speed of less than ${\frac{1}{2}\theta}_{1}$ are labelled as invalid: $M_{v}\left[ x,y \right]=0.$

Conditions based on greyscale intensity fluctuations: the exact same validity condition, as has just been described for the variance in optical flow speed, is applied to the variance of the intensity fluctuations.

Another condition concerns the total power contributed by all the frequencies within the bandwidth of the CBF-peak $\left[ f_{1},f_{2} \right]$ . Specifically, a pixel is classified as valid, if its total power contributed by the frequency band $\left[ f_{1},f_{2} \right]$ is at least $\theta\cdot$100% of the total power contributed by the bandwidth of the main peak within the spatially averaged PSD:

$$\sum_{f_{1}}^{f_{2}} \hat{P}_{x,y}\left( f \right)\geq\theta\cdot\sum_{f_{1}}^{f_{2}} \left\langle\hat{P}_{x,y}\left( f \right) \right\rangle_{x,y}$$

Where $\theta$ is set to 0.15.

Furthermore, to classify a pixel as valid, it is required that the peak frequency is lower than the upper bound of the frequency band designating the first higher harmonic.

Finally, it is also required that the maximum of a pixel’s PSD $\hat{P}_{x,y}\left( f \right)$ within the CBF-band $\left[ f_{1},f_{2} \right]$ has an amplitude, which is at least 50% higher than the minimum of $\left\langle\hat{P}_{x,y}\left( f \right) \right\rangle_{x,y}$ within $\left[ f_{1},f_{2} \right]$.

If one or more of the above-listed conditions are not satisfied, the pixel is classified as invalid, and its frequency is treated as a missing value in the activity map (i.e. set to NaN) and in the subsequent analysis pipeline.

The active percentage is simply defined as the ratio of the number of valid pixels to the total number of pixels covered by the entire field of view.

2.3. Autocorrelation of the activity map

In accordance with the notation used in the previous section, we denote the activity map by $\mathbf{A}\left[ x,y \right]$ and the binary validity mask by $\mathbf{M}_{\mathbf{v}}\left[ x,y \right]$.

In a first step, the matrix $\mathbf{A}$ gets centred:

$$\mathbf{A}_{\mathbf{c}}\left[ x,y \right]=\mathbf{A}\left[ x,y \right]-\left\langle\mathbf{A}\left[ x,y \right] \right\rangle_{x,y}\cdot\mathbf{J}_{N,M}$$

where $\mathbf{A}_{\mathbf{c}}$ denotes the centered activity map and $\mathbf{J}_{N,M}$the $N\times M$ matrix containing just ones.

The mean value of the activity map $\left\langle\mathbf{A}\left[ x,y \right] \right\rangle_{x,y}$is calculated by ignoring invalid pixels, which can formally be written as:

$\left\langle\mathbf{A}\left[ x,y \right] \right\rangle_{x,y}=\frac{1}{N_{v}}\cdot\sum_{x,y} \mathbf{A}\left[ x,y \right]\circ\mathbf{M}_{\mathbf{v}}\left[ x,y \right]$ ,

Where “$\circ$“ denotes the element-wise product and $N_{v}$ the total number of valid pixels. Furthermore, invalid pixels in $\mathbf{A}_{\mathbf{c}}$ are set to zero.

Next, we zero-pad the centred activity map $\mathbf{A}_{\mathbf{c}}$ in such a way that the zero-padded activity map, which we denote by $\mathbf{A}_{\mathbf{c+0}}$, has twice the size along each dimension ($2N\times2M$). The zero-padding can formally be denoted as:

$$\mathbf{A}_{\mathbf{c+0}}=\mathbf{A}_{\mathbf{c}}\oplus\mathbf{0}_{N,M}$$

Where $\oplus$ denotes the direct sum and $\boldsymbol{0}_{N,M}$ the zero-matrix of dimension $N\times M$. By using the Wiener-Khinchin theorem, we compute a first biased version of the autocovariance of the activity map, which is biased due to the zero-padding as well as possible missing values (invalid pixels in the activity map):

$$\mathbf{C}_{\mathbf{b}}\left[ \Delta x,\Delta y \right]=\left\langle\mathbf{A}_{\text{c+0}}\left[ x,y \right],\mathbf{A}_{\text{c+0}\left[ x+\Delta x,y+\Delta y \right]} \right\rangle=\mathcal{F}_{2}^{-1}\left[ \left| \mathcal{F}_{2}\left〖 \left( \mathbf{A} \right〗_{\text{c+0}} \right) \right|^{2} \right]$$

Where $\mathcal{F}_{2}$ denotes the two-dimensional Fourier transform.

To correct for the zero-padding as well as for the missing values, we repeat the above process for the validity mask:

$$\mathbf{N}_{\mathbf{v}}=\mathcal{F}_{2}^{-1}\left[ \left| \mathcal{F}_{2}\left( \mathbf{M}_{\mathbf{v+0}} \right) \right|^{2} \right]$$

Renormalizing the biased autocovariance with an element-wise division by $\mathbf{N}_{\mathbf{v}}$ yields the autocovariance of the activity map:

$\mathbf{C}^{\mathbf{'}}=\mathbf{C}_{\mathbf{b}}\frac{}{}\mathbf{N}_{\mathbf{v}}$.

An element-wise division of the latter expression by the variance of $\mathbf{A}_{\mathbf{c}}\left[ x,y \right]$ finally yields the (two-dimensional) autocorrelation of the activity map, which is free from wrap-around artifacts (thanks to the zero-padding) and uses an unbiased correction for missing values.

2.4. Space-time correlations

As mentioned in the main text, for the analysis of the local wave field, the dynamically filtered and possibly otherwise preprocessed video is divided into square videos. Each of these new videos has a side length of 3.5 times the spatial correlation length. Subsequently, a separate space-time correlation is calculated for each of these videos. To determine the wave propagation velocity the following computational steps are performed for each partial video.

For each partial video, the space-time correlation $C\left( \Delta x, \Delta y, \Delta t \right)$ is calculated for only three time lags ($\Delta t=0$, 1, 2 [FPS^-1^]). The time lag is therefore chosen explicitly, whereas in the spatial domain, the spatial cross-correlation between two frames separated by the time lag Δ*t* is computed by using the Wiener-Khinchin theorem. Once again, the computation takes care of the wrap-around artifact by using zero-padding in the spatial domain and subsequent correction for proper normalization of the cross-correlation. Note that for a single time lag Δ*t* the cross-correlation is averaged over all consecutive frames separated by Δ*t*, i.e. $t=0,1,2,\ldots, N_{t}$–Δ*t*–$1$. Subsequently, for consecutive time lags, the peak in the cross-correlogram gets fitted by a two-dimensional Gaussian and is tracked and averaged over the available time lags, assigning a wave propagation velocity to each subvideo. Finally, the wave propagation velocity is averaged over all available subvideos to determine the average wave speed.
